# Continuous infusion of PTH_1–34_ delayed fracture healing in mice

**DOI:** 10.1038/s41598-018-31345-1

**Published:** 2018-09-04

**Authors:** Kiminori Yukata, Tsukasa Kanchiku, Hiroshi Egawa, Michihiro Nakamura, Norihiro Nishida, Takahiro Hashimoto, Hiroyoshi Ogasa, Toshihiko Taguchi, Natsuo Yasui

**Affiliations:** 1 Department of Orthopedics, Institute of Health Biosciences, The University of Tokushima Graduate School, Tokushima, Japan; 2 Department of Orthopedic Surgery, Yamaguchi University Graduate School of Medicine, Yamaguchi, Japan; 3 Department of Organ Anatomy, Yamaguchi University Graduate School of Medicine, Yamaguchi, Japan

## Abstract

Hyperparathyroidism, which is increased parathyroid hormone (PTH) levels in the blood, could cause delayed or non-union of bone fractures. But, no study has yet demonstrated the effects of excess continuous PTH exposure, such as that seen in hyperparathyroidism, for fracture healing. Continuous human PTH_1–34_ (teriparatide) infusion using an osmotic pump was performed for stabilized tibial fractures in eight-week-old male mice to determine the relative bone healing process compared with saline treatment. Radiographs and micro-computed tomography showed delayed but increased calcified callus formation in the continuous PTH_1–34_ infusion group compared with the controls. Histology and quantitative histomorphometry confirmed that continuous PTH_1–34_ treatment significantly increased the bone callus area at a later time point after fracture, since delayed endochondral ossification occurred. Gene expression analyses showed that PTH_1–34_ resulted in sustained *Col2a1* and reduced *Col10a1* expression, consistent with delayed maturation of the cartilage tissue during fracture healing. In contrast, continuous PTH_1–34_ infusion stimulated the expression of both *Bglap* and *Acp5* through the healing process, in accordance with bone callus formation and remodeling. Mechanical testing showed that continuously administered PTH_1–34_ increased the maximum load on Day 21 compared with control mice. We concluded that continuous PTH_1–34_ infusion resulted in a delayed fracture healing process due to delayed callus cell maturation but ultimately increased biomechanical properties.

## Introduction

Endogenous parathyroid hormone (PTH), which is secreted from the parathyroid glands, controls the calcium levels in the blood, and elevation of serum PTH stimulates the conversion of 25-hydroxy vitamin D into 1,25-dihydroxy vitamin D^1–3^ and increased renal calcium retention in the kidney^4,5^. The active form of vitamin D also enhances intestinal calcium absorption. Furthermore, PTH directly enhances osteoclastic bone resorption, resulting in the release of calcium into the blood stream from the skeleton via binding to osteoblasts^6^. Thus, excess continuous PTH exposure, such as that seen in hyperparathyroidism, causes osteopenia or osteoporosis in humans.

The paradoxical effects of PTH on bone metabolism resulted in increased metaphyseal bone in rats given a daily injection of parathyroid extract in the 1930s^7^. In 1970, Kalu *et al*. also demonstrated the anabolic effect on the bone of 20-day daily subcutaneous injection of low doses of both parathyroid extracts and pure PTH, resulting in an increased bone mineral density (BMD) in the rat femoral metaphysis^8^. These data suggested that intermittent low doses of human PTH might increase the BMD for patients with osteoporosis. In 1976 and 1980, Reeve *et al*. reported a successful clinical trial of once-daily human PTH_1–34_ therapy for postmenopausal women with osteoporosis^9,10^. Human PTH_1–34_ or _1–84_ is already used to increase the bone mass in osteoporotic patients as an anabolic agent after prospective, randomized, multicenter clinical studies^11,12^.

Given the successfully documented bone anabolic effects of PTH in humans, intermittent PTH treatment for bone healing has recently been investigated in several experimental and clinical studies^13–18^. Bone injury results in the proliferation of the periosteal progenitor population with the accumulation of the expanded progenitor population along the bone surface^19,20^. These cells subsequently undergo differentiation into both bone and cartilage tissues. Chondrocytes formed at the fracture site hypertrophy, the cartilage matrix undergoes calcification, and the process of endochondral ossification is completed with vascularization and primary bone formation on the cartilage template. Intramembranous ossification occurs in parallel with endochondral ossification and involves the direct differentiation of the periosteal progenitors into osteoblasts and bone formation. Fractures are healed when calcified callus tissue unites the bone fragments at the fracture site, although remodeling of the fracture with formation of a more organized bone structure (lamellar bone) continues^19^. According to previously published animal studies, intermittent subcutaneously administration of PTH_1–34_ increased the callus volume via progenitor cell proliferation, which may enhance fracture healing^14,15,18,21^. Clinical studies have also indicated that both PTH_1–34_ and _1–84_ accelerated fracture healing in the distal radius and pubis^16,17^. On the other hand, some clinical reports suggested that hyperparathyroidism, which is increased parathyroid hormone (PTH) levels in the blood, could cause delayed or non-union of bone fractures^22–24^. The effects of continuous PTH exposure for bone fracture healing have not been yet elucidated.

In order to better understand the PTH_1–34_ therapy for bone healing, we therefore examined the effects of continuous PTH_1–34_ infusion, a regimen in stark contrast to the traditional intermittent administration, which by the hormone is removed from the plasma within 4 hours after subcutaneous injection^25^, on the radiographic, histologic, and biomechanical properties of mouse tibial fracture healing.

## Results

### Continuous PTH infusion delayed the bone callus formation

Histomorphometric and micro-CT evaluations for the contralateral tibiae demonstrated that continuous PTH_1–34_ infusion for two weeks caused osteopenia, and intermittent administration increased the bone mass, although bone formation and resorption were enhanced in both PTH-treated groups (Supplemental Tables 1 and 2). These findings are consistent with the past data about the bone response to exogenous continuous or intermittent PTH administration^25–27^.

**Figure 1:**
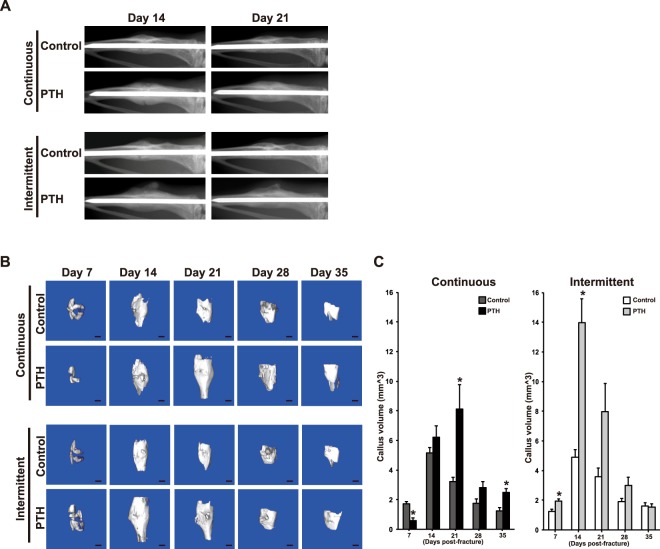
Continuous treatment of PTH_1–34_ delayed calcified callus formation. (**A**) Serial radiographs of representative mice continuously and intermittently treated with normal saline or PTH_1–34_ at 14 and 21 days post-fracture. PTH_1–34_ enhanced fracture callus formation in both the continuous and intermittent treatment groups. However, at day 14, the fracture line is clearly visible in only the continuous PTH_1–34_ treatment group. (**B**) Fractured specimens were harvested, and high-resolution micro-CT was performed on specimens continuously and intermittently treated with normal saline or PTH_1–34_ (n = 5, Scale bar: 1 mm). Representative scans are shown for days 7, 14, 21, 28, and 35 for the calcified external callus. (**C**) The mean calculated external bony callus volume was quantified. Continuous PTH_1–34_ treatment delayed the external bony callus compared to the control specimens. Statistical significance is denoted as follows: ^★^*p* < 0.05 compared to each control.

The effects of continuous and intermittent PTH_1–34_ treatments on fracture healing were compared by radiography and micro-CT. Soft X-rays were obtained 14 and 21 days following fracture. A radiolucent line at the fracture site persisted in fractures from the continuous PTH infusion group at day 14 following fracture, whereas bone union was achieved in the control group (Fig. 1A). In contrast, PTH_1–34_ enhanced radiographic evidence of fracture callus formation in an intermittent treatment, and bone union was observed in the fractures of intermittently PTH-treated mice by Day 14. Radiographic assessment of union and delayed union rates demonstrated that 14 (93.3%) mice in the control group achieved bone union at 2 weeks compared with 5 (33.3%) continuously PTH-treated mice (Table 1; *p* < 0.05). In the control group, delayed union was observed in only 1 (6.7%) mouse, while in the continuous PTH treatment group delayed union was observed in 10 (66.7%) mice (*p* < 0.05). No nonunion was seen in all experimental groups.

**Table 1:** Union and delayed union rate by radiographic assessment at 2 week after fracture.

	Union	Delayed union
**Continuous groups**		
Control	14/15 (93.3%)	1/15 (6.7%)
PTH	5/15 (33.3%)*	10/15 (66.7%)*
**Intermittent groups**		
Control	14/15 (93.3%)	1/15 (6.7%)
PTH	15/15 (100%)	0/15 (0%)

*p < 0.05 compared with the control group.

Micro-CT was performed on tibiae harvested at 7, 14, 21, 28 and 35 days following fracture in order to quantify the amount of external calcified fracture callus tissue formed (Fig. 1B,C). At 7 and 14 days, the calcified callus volume was significantly higher (1.52-fold and 2.85-fold, respectively; *p* < 0.05) in fractures from intermittently PTH-treated mice than in intermittently vehicle-treated mice (Fig. 1C). The calcified callus volume peaked at 14 days in these two groups. In contrast, calcified callus formation was significantly decreased at 7 days (0.34-fold; *p* < 0.05) in continuously PTH-treated mice compared to continuously vehicle-treated mice, but ultimately increased at 21 days (2.53-fold; *p* < 0.05). The callus formation peaked at 21 days in continuously PTH-treated mice after one week lag compared to continuously vehicle-treated control, indicating delayed bone callus formation. There was no significant difference between 2-week control and 3-week PTH groups with continuously treated in the amount of calcified callus volume at the peak (*p* = 0.145). Remodeling occurred after peak callus formation but was also delayed in continuously PTH-treated compared to control mice. At 35 days, fractures in continuously PTH-treated mice were still undergoing remodeling, resulting in increased mineralized callus at this point compared to control mice (1.98-fold; *p* < 0.05). Thus, the peak callus formation was delayed in continuously PTH-treated mice compared to the other groups. Enhanced bone regeneration did not occur in mice treated by continuous PTH_1–34_ administration, although a more robust effect for calcified callus volume was observed with intermittent treatment.

### Continuous PTH_1–34_ infusion increases the biomechanical strength of fractured tibiae

**Figure 2:**
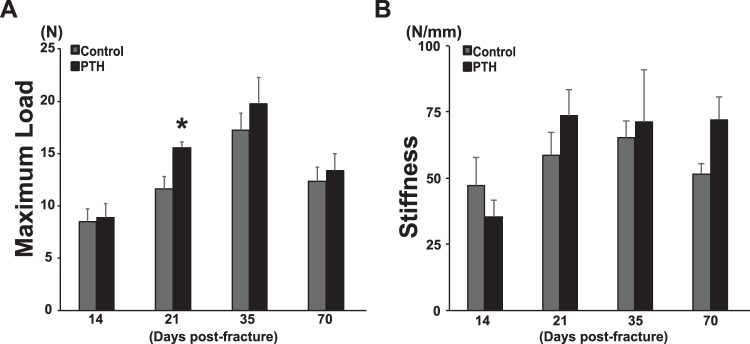
Continuous PTH_1–34_ treatment increased the biomechanical properties in mouse tibial fractures. Fractured tibiae were retrieved upon animal sacrifice at 14, 21, 35, and 70 days and tested using a three-point bending machine to determine the maximum load (**A**) and stiffness (**B**). Continuous PTH_1–34_ treatment improved the biomechanical properties of fractured tibiae at 21 days post-fracture. Statistical significance is denoted as follows: ^★^*p* < 0.05 compared to each control.

Since biomechanical testing is considered a definitive measure of bone union, the three-point bending test was performed on fractured tibiae harvested from control and PTH_1–34_ continuously treated mice at 14, 21, 35 and 70 days. The maximum load was significantly elevated by continuous PTH_1–34_ infusion compared to vehicle control at only 21 days (1.37-fold; *p* < 0.05) (Fig. 2A). PTH_1–34_ also stimulated increases in stiffness at 21 (1.26-fold) and 70 days (1.40-fold), although statistical significance was not achieved when compared to the vehicle control mice (Fig. 2B). These biomechanical properties were consistent with the micro-CT results that showed increased bone callus tissue in continuously PTH-treated mice (Fig. 1B,C).

### Continuous PTH_1–34_ infusion increases both the cartilage and bone callus formation but delays endochondral bone formation

**Figure 3:**
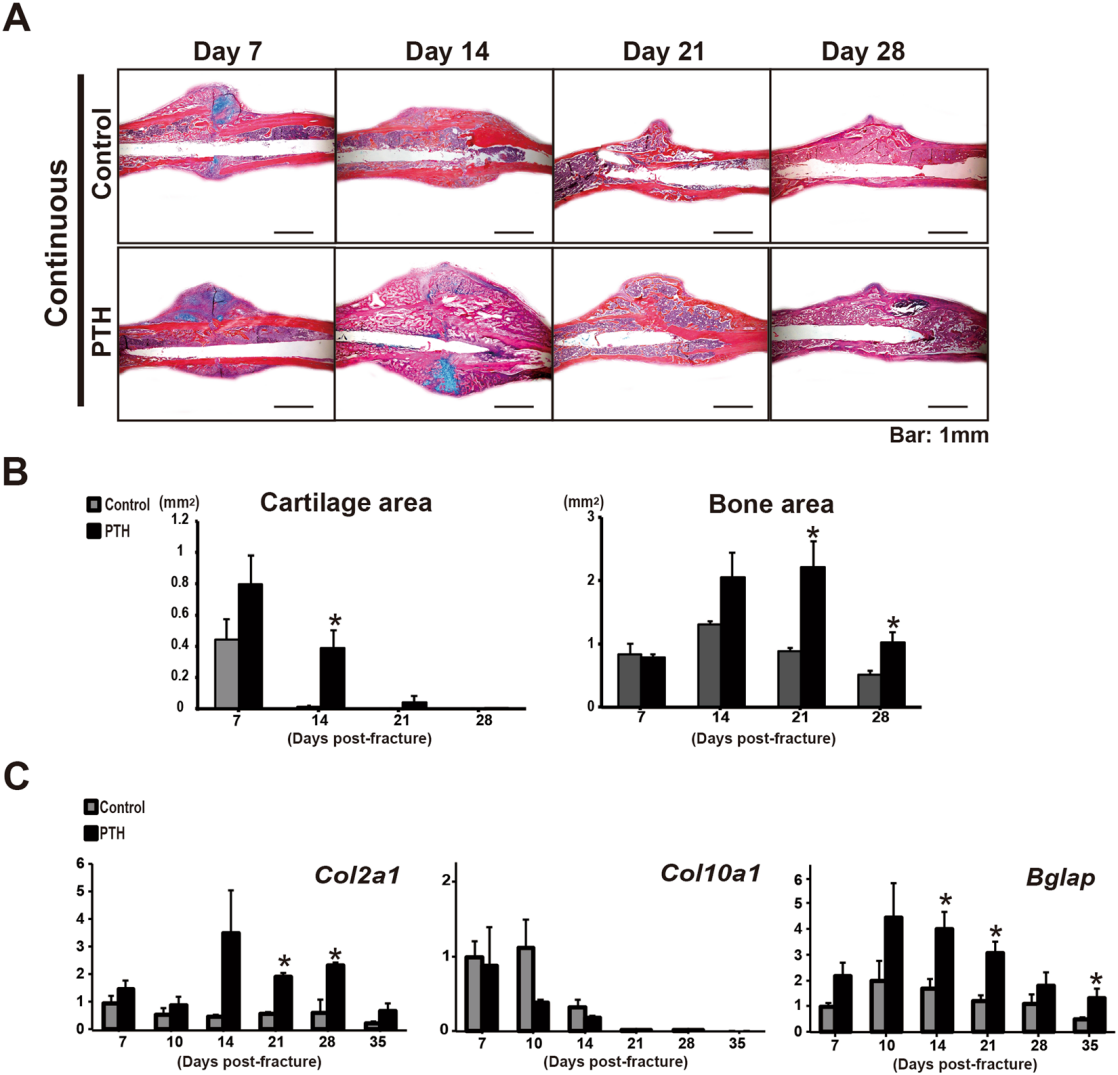
Continuous PTH_1–34_ administration increased both the cartilage and bone area and delayed cartilaginous callus maturation. Representative alcian blue hematoxylin/orange G eosin sections of samples continuously treated with normal saline or PTH_1–34_ at 7, 14, 21, and 28 days post-fracture are shown (**A**, Scale bar: 1 mm). Histology showed that PTH_1–34_ enhanced both cartilaginous and bony callus formation. However, PTH-treated mice showed a delayed completion of endochondral bone formation at 14 days post-fracture compared with control mice. Histomorphometry showed a significant increase in the cartilage and bone areas by PTH_1–34_ treatment. (**B**) Real-time RT-PCR analyses were performed using RNA collected from fracture calluses with normal saline or PTH continuous treatment on days 7, 10, 14, 21, 28, and 35 post-fracture. (**C**) A gene expression analysis showed an increase in *Col2a1* expression and a decrease in *Col10a1* expression, suggesting delayed cartilage maturation. In contrast, the expression of *Bglap* was increased during fracture healing in continuously PTH_1–34_ -treated fractures. Statistical significance is denoted as follows: ^★^*p* < 0.05 compared to each control treatment group.

A detailed temporal analysis of the histology and gene expression was performed on fractured tibiae in order to determine whether or not continuous PTH_1–34_ treatment affected the cartilage and bone callus formation during bone repair. Quantitative histomorphometry confirmed the micro-CT findings and showed that continuous PTH_1–34_ treatment significantly increased bone callus area at days 21 and 28 after fracture, but delayed the callus formation process (Fig. 3A,B). PTH_1–34_ also significantly increased the cartilage callus area in continuously PTH-treated mice only at 14 days post-fracture (Fig. 3B). However, the existence of this residual cartilage is likely due to the ability of PTH_1–34_ to promote chondrocyte proliferation and inhibit terminal differentiation.

To determine the effect of continuous PTH_1–34_ infusion on chondrogenesis and osteogenesis during fracture healing, we examined and compared the expression of related genes over time. Real-time reverse transcription (RT)-PCR showed that the peak levels of *Col2a1* (a marker of immature chondrocytes) gene expression were higher in the fractures of PTH-treated mice than in those of vehicle-treated mice throughout the healing process, being statistically significant at 21 and 28 days post-fracture, whereas the *Col10a1* (a marker of mature chondrocytes) gene expression was decreased in the PTH-treated group at 10 and 14 days post-fracture (Fig. 3C). These results suggest a delay in the chondrocyte maturation secondary to continuous PTH_1–34_ treatment. The temporal expression of the osteoblast gene *Bglap* (*Osteocalcin*; a marker of mature osteoblasts) was also examined in the healing fractures to determine the effects of continuous PTH_1–34_ treatment on bone formation in calluses. The *Bglap* expression peaked at 10 days in both PTH-treated and vehicle-treated mice and was significantly increased at 14, 21, and 35 days compared to the baseline. This increase in *Bglap* expression is consistent with an increased mineralized callus and delayed maturation of the osteoid matrix. These findings suggest that the delayed increase in bone callus by the continuous administration of PTH_1–34_ is primarily due to the delayed ossification of the increased cartilage and pre-mature bone callus.

### Continuous PTH_1–34_ infusion induces osteoclastic callus remodeling during fracture healing

**Figure 4:**
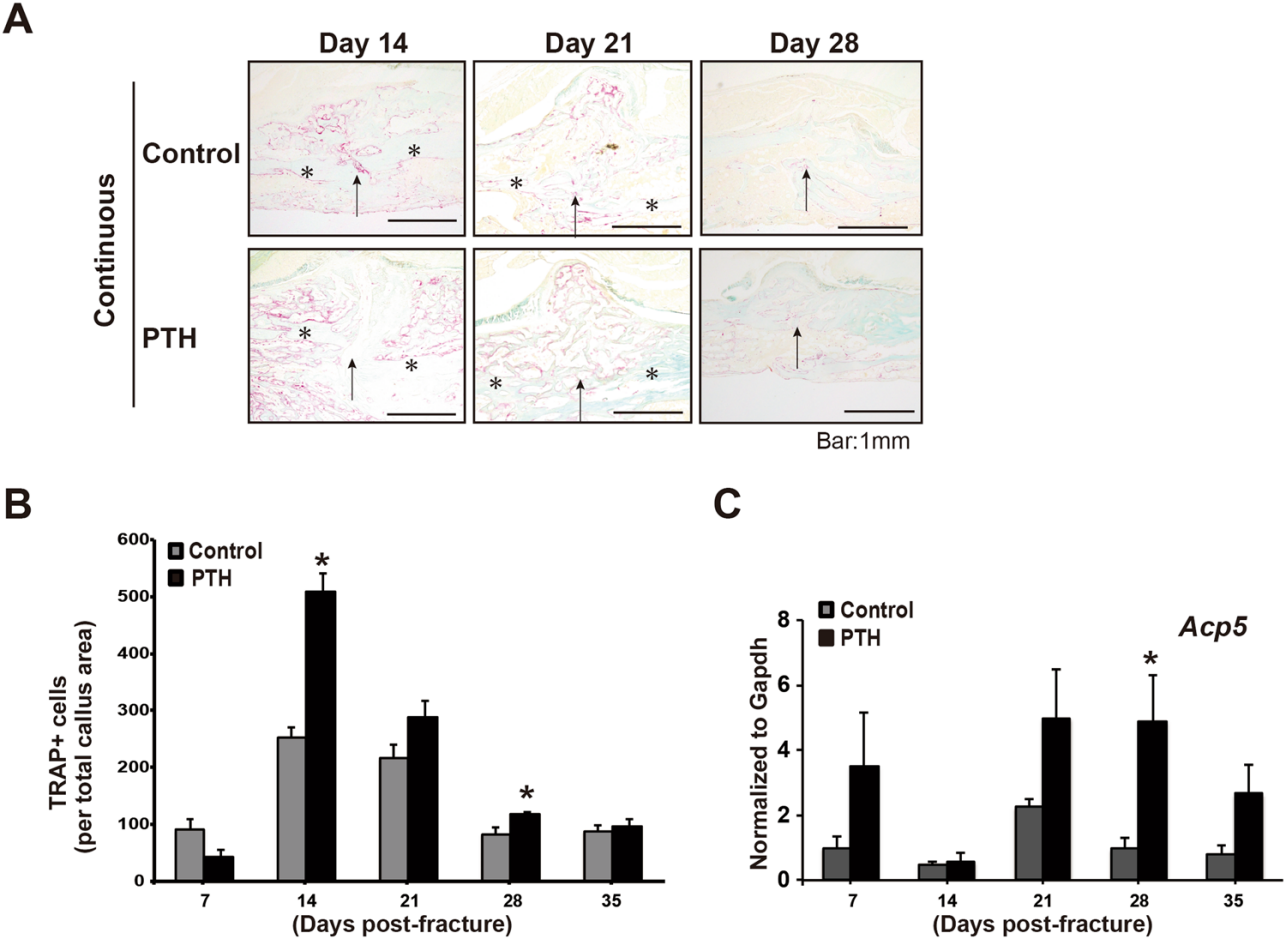
The effect of PTH_1–34_ on osteoclast formation during fracture healing. Representative TRAP staining sections of fractures continuously treated with normal saline or PTH_1–34_ at days 14, 21, and 28 after fracture are shown (**A**, *the original cortex bone, Arrow: the fracture site, Scale bar: 1 mm). TRAP-positive cells numbers are shown. (**B**) The time course change of *Acp5* gene expression is measured by real-time RT-PCR. (**C**) Statistical significance is denoted as follows: ^★^*p* < 0.05 compared to each control.

To determine the effects of continuous PTH treatment on osteoclastic bone resorption during fracture healing, TRAP staining and quantitative RT-PCR were performed to quantify the osteoclast numbers and *Acp5* (*Tartrate-resistant acid phosphatase*: a marker of osteoclasts) gene expression in callus tissues at 7, 14, 21, 28, and 35 days post-fracture (Fig. 4). The number of TRAP-positive cells was significantly larger in PTH-treated mice than in vehicle-treated mice at 14 and 28 days post-fracture (Fig. 4B). Interestingly, real-time RT-PCR revealed that PTH did not affect the *Acp5* expression during the PTH administration period but did increase the gene expression at the later stage (Fig. 4C). These data suggest that increased osteoclasts in PTH-treated fractures are an indirect effect caused by larger mineralized callus formation rather than a direct effect of the drug.

## Discussion

The present study showed that the callus formation and remodeling was significantly delayed due to the impairment of intramembranous and endochondral bone formation by continuous PTH_1–34_ infusion. However, higher biomechanical properties were ultimately achieved in continuously PTH-treated fractures compared to vehicle-treated controls. It is known that hyperparathyroidism could clinically result in delayed fracture union, and healing accelerated after excision of a parathyroid adenoma^22–24^. Recently, Liu *et al*. reported that secondary hyperparathyroidism due to chronic kidney disease impaired calvarial and femoral bone defect healing in rats^28^. These data indicated that continuous PTH exposure impaired bone fracture healing.

In 1999, Andreassen *et al*. first reported that once-daily intermittent PTH administration significantly increased the calcified callus volume and mechanical strength of the fractured bone^13^. Further experimental animal studies with daily systemic PTH injections also resulted in similar outcomes^14,18^. However, the appropriate interval and dose of PTH administration on bone fracture healing have been unclear, and no study has examined the effects of continuously given PTH. Our study showed that continuous PTH_1–34_ infusion improved early cartilaginous callus formation in the fracture callus. This finding is consistent with several previous reports that describe increases in the cartilaginous callus volume and elevation of chondrogenic genes expression due to intermittent PTH administration, suggesting enhanced chondrogenesis during fracture healing^15,21^. Similar to fracture healing, continuous PTH administration increased the width of the epiphyseal plate via enhanced chondrocyte proliferation in the growth plate^29^. *In vitro* experiments have also shown that PTH accelerated the proliferation of chondrocytes^30,31^ and affected matrix production by growth plate chondrocytes, thereby stimulating aggrecan synthesis^32^. On the other hand, the present study indicated that continuous PTH administration delayed endochondral bone formation. PTH/PTH-related peptide (PTHrP) is considered to inhibit chondrocyte hypertrophy^33–35^. In Jansen-type metaphyseal chondrodysplasia, the expression of a constitutively active mutant PTH/PTHrP receptor limited the numbers of chondrocytes that hypertrophied, thereby controlling the rates of endochondral bone formation^36^. Our previous studies using the same mouse tibial fracture model showed that intermittent treatment of PTH_1–34_ also delayed cartilage callus maturation^18,37^. These data indicate that exogenous PTH administration may therefore enhance chondrogenesis but suppress the hypertrophy of chondrocytes and delay the replacement of cartilage with bone during fracture healing. In contrast, other studies using a rat closed femoral fracture model showed that PTH treatment enhanced the rate of chondrocyte maturation and mineralization, as indicated by both an earlier peak in Sox5 and type X collagen genes expression^15,21^. The discrepancies in these results might be influenced by the instability of the fracture site and the degree of periosteal damage due to differences in the animal model.

Previous *in vivo* studies have shown that intermittent PTH treatment increased the osteoblast numbers in adult rats with few proliferating osteoblast progenitors by stimulating the differentiation of quiescent bone surface cells, suggesting that the PTH-mediated increase in bone formation was due to the activation of bone lining cells^38,39^. Intermittent PTH treatment also increased the population of proliferative cells among implanted bone marrow stromal cells^40^ and increased early osteoblast proliferation *in vitro* with elevated cyclinD1 levels in *in vivo* BMSC implants treated with PTH^41^. Nakajima *et al*. and Yukata *et al*. showed that the number of PCNA-positive subperiosteal osteoprogenitor cells was significantly increased in the calluses of intermittently PTH-treated fractures^14,18^. A number of *in vitro* studies have been conducted to determine the mechanism underlying the effects of PTH on the proliferation and differentiation of osteoblasts, but the results obtained have been inconsistent. PTH stimulated the proliferation of primary osteoblastic cells isolated from human trabeculae and chick calvariae^42,43^, but inhibited the proliferation of the rat osteoblastic cell line UMR-106^44,45^. PTH suppressed the alkaline phosphatase activity in the rat osteoblastic cell line ROS-17/2^46^ but had a stimulatory effect in the mouse osteoblastic cell line MC3T3-E1^47^. PTH inhibited bone nodule formation by primary osteoblastic cells isolated from calvariae of rat embryos^48^. Several researchers have reported that PTH exerted diverse effects on osteoblast differentiation, depending on the differentiation stage or cell type^49–51^. Ishizuya *et al*. showed that continuous PTH exposure inhibited the differentiation of osteoblastic cells isolated from rat calvariae by suppressing the ALP activity, but intermittent exposure exerted an anabolic effect on osteoblast differentiation, depending on the timing of exposure^52^. Taken together, action of promoting immature callus cells proliferation and inhibiting differentiation is more pronounced in fractures treated with continuous PTH_1–34_ administration than in those treated with intermittent administration.

Experiments using intact and osteopenic animals have demonstrated that intermittent administration of PTH increases the bone mass, whereas the continuous infusion causes osteopenia^25–27^. Podbesek *et al*. and Tam *et al*. reported that daily injection of PTH significantly increased the rates of bone formation and resorption as well as the iliac trabecular bone volume, whereas continuous infusion resulted in no increase in the trabecular bone volume because of increased osteoclastic surfaces and less increased osteoblastic surfaces in rats and dogs^25,26^. In addition, continuous infusion of PTH results in hypercalcemia in mice and produces a model of primary hyperparathyroidism, while intermittent PTH administration only results in a small and transient increase in calcium^53^. These findings are consistent with those of a previous study in primary hyperparathyroidism with the continuous administration of PTH, which is known to induce osteoclastic bone resorption^54–56^. In our study, we observed significant differences in osteoclast marker gene (*Acp5*) expression at a later time point during fracture healing, whereas the osteoclast number around the fracture site was largest at two weeks after fracture in the continuously PTH-treated group. The increase in the number of osteoclasts seemed to have been accompanied by an increase of bone callus formation at two and three weeks post-fracture, rather than a direct effect of PTH for osteoclastogenesis.

At present, limited evidence is available regarding the effect of teriparatide in fracture healing in humans. Despite the fact that intermittent PTH dose-dependently increased the callus volume in the animal studies^13,14,18^, a placebo-controlled trial of distal radius fractures in postmenopausal women showed that 20 μg/day teriparatide shortened the time of cortical bridging on CT but surprisingly failed to accelerate the time to radiologic healing at a higher dose of 40 μg/day^16^. However, a subgroup analysis showed that the group with the higher dose had richer, more calcified callus formation on radiographs than the low-dose group^57^. These findings therefore suggest that increasing the amount of PTH and the frequency of administration promotes callus formation but may delay callus maturation on evaluation by X-ray or CT.

Several limitations associated with the present study warrant mention. We indirectly evaluated whether or not continuous and intermittent treatment worked well using bone histomorphometric analyses and micro-CT but did not perform direct assessments, such as measuring the PTH concentration in the blood. No consideration is given to the responsiveness of target cells during intermittent or continued exposure to PTH. Furthermore, sham surgeries (pump implantation and removal) were not performed in the intermittent treatment groups, so these findings cannot be compared directly with the continuous groups. We histologically examined endochondral and intramembranous ossification, but their distinction was not clear. In order to clarify this distinction, a cell fate analysis divided into chondrocyte and osteoblast lineages using molecular biological techniques should be performed. The use of genetically modified or disease specific model mouse, or PTH (1–84) might be more suitable to elucidate bone fracture healing in patients with hyperparathyroidism, rather than exogenous recombinant PTH_1–34_ (teriparatide) infusion. Finally, since gender-specific differences in the skeletal response to continuous PTH have been identified, fracture healing in women should also be verified^58^.

## Conclusions

We determined that continuous exogenous PTH_1–34_ administration markedly delayed the callus maturation and remodeling, while intermittent treatment has positive effects on the fracture callus volume and healing. This study provides important findings concerning the delayed fracture healing of the patient with primary or secondary hyperparathyroidism, and might improve our understanding of the appropriate interval of PTH administration for bone fracture management. Further studies are required to elucidate the optimum dose or treatment interval to promote fracture healing using PTH_1–34_.

## Methods

### Experimental animals and surgery

Healthy 8-week-old male C57BL/6J mice were obtained from CLEA Japan, Inc. (Tokyo, Japan) and housed 5 per microisolator cage in a vivarium housing room on a 12-hour light/dark cycle and provided a free access to a diet. A mouse tibia fracture model has been used in previous studies^18,37^. In brief, after general anesthesia with isoflurane, a 5-mm longitudinal incision was made at the front of the right proximal tibia. An open bone fracture was created with a No. 11 scalpel blade at the proximal third of the tibial diaphysis. We tried not to break the fibula. An intramedullary pin 26G Quincke type spinal needle (Nippon BD Company, Ltd., Tokyo, Japan) was introduced into the tibial canal through a tibial tuberosity. The wound was closed using nylon sutures. Animal use and all methods were approved by the Laboratory Animal Care and Use Committee of The University of Tokushima Graduate School (No. 12011). All animal experiments were performed in accordance with the relevant guidelines and regulations.

#### Experiment 1

To confirm whether or not two administration (intermittent or continuous) regimens worked in the current study, mice were randomly divided into four treatment groups after fracture surgery: recombinant human PTH_1–34_ and normal saline continuous and intermittent treatment. The mice in the 2 continuous groups were infused with a saline or recombinant human PTH_1–34_ (Forteo; 40 μg/kg/day) for 2 weeks using Alzet miniosmotic pump 2002 (Muromachi Kikai Co., Ltd., Tokyo, Japan) implanted in the dorsal subcutaneous tissue. Each pump was removed at two weeks after surgery. For the mice in the 2 intermittent groups, saline or PTH (40 μg/kg) was administered subcutaneously from the day of fracture surgery once a day for 2 weeks. The total amount of saline and PTH_1–34_ administered was the same for both the infusion and daily injection groups. Two weeks after surgery, the contralateral unfractured tibiae were extracted for bone histomorphometry and micro-CT analyses (n = 5 per group). In addition, radiologic analyses for the fractured tibiae were performed to clarify the bone callus formation on days 7, 14, 21, 28, and 35 after fracture in the 4 groups (n = 5 per group).

#### Experiment 2

We focused the continuous groups in this experiment. To assess the differences between continuous saline and PTH_1–34_ infusions during fracture healing, histology/histomorphometry, gene expression, and biomechanical analyses were performed.

### Bone histomorphometry for the contralateral unfractured tibiae

Mice were subcutaneously injected with calcein (20 mg/kg body weight) on days 1 and 4 prior to being killed at 2 weeks after fracture. The left unfractured tibiae were excised, fixed with 70% ethanol after the removal of the soft tissues After micro-CT evaluation, the samples were embedded in glycol methacrylate, and sections were cut at 3 μm with toluidine blue. Bone histomorphometry was performed on undecalcified sections with the Villanueva staining. We defined the regions of interest as the area between approximately 0.3 mm and 1.125 mm proximal to the growth plate in the proximal tibia, including the secondary trabecular spongiosa. Bone morphometric parameters were calculated using the OsteoMeasure software program (OsteoMetrics, Inc., Decatur, GA, USA). These included bone volume per tissue volume (BV/TV; %), trabecular thickness (Tb.Th; μm), trabecular number (Tb.N; /mm), and trabecular separation (Tb.Sp; μm) for bone architectural parameters; osteoid volume per bone volume (OV/BV; %), osteoid surface per bone surface (OS/BS; %), osteoblast surface per bone surface (Ob.S/BS; %), osteoid thickness (O.Th; μm), mineralizing surface per bone surface (MS/BS; %), mineral apposition rate (MAR; μm/day), and bone formation rate per bone surface (BFR/BS; μm^3^/μm^2^/day) for bone formation parameters; and eroded surface per bone surface (ES/BS; %), osteoclast surface per bone surface (Oc.S/BS; %), and osteoclast number per bone surface (N.Oc/BS; /100 mm) for bone resorption parameters.

### Radiographic assessment of fracture healing

Soft X-ray images were taken using a soft X-ray apparatus (CMB-2; SOFTEX, Tokyo, Japan) at the time of surgery and weekly following surgery until sacrifice. Radiographs of the fractured tibiae taken at 2 weeks were used to assess either bone union or delayed union. Each callus on the two cortices was evaluated by three orthopaedic surgeons blinded to the group. Bone union was defined as when two of the two cortices and/or external calluses were bridged, and delayed union was defined as when either side of the cortical bone or callus was not bridged.

### Micro-computed tomography analyses for fractured and unfractured tibiae

Fractured and contralateral unfractured tibiae were harvested at indicated times and scanned using a micro-CT system (Latheta LCT-200; Hitachi, Ltd. Healthcare BU, Tokyo, Japan). An integration time of 13 ms, a current of 500 μA, and an energy setting of 50 kVp, axial field of view; 24 mm, with an isotropic voxel size of 24 μm were used. A threshold density was set at 160 mg/cm^3^. The original cortical bone with bone marrow was removed by contouring around the external callus and along the edge of the cortical bone using a 2D evaluation of several slices in the transverse anatomic plane so that only the mineralized callus was identified. Volume-rendered three-dimensional CT images were reconstructed using the Osirix Lite software program (PIXMEO, Inc., Bernex, Switzerland). Calibration of the density range was performed with a manufacturer-provided phantom. We used 5 specimens for each group. Following micro-CT imaging of the bone, the fractured tibiae were frozen and stored at −20 °C until the mechanical testing.

### Histology and histomorphometric analyses for fractured tibiae

Fractured tibiae were harvested and processed for histology. Mice were sacrificed at 7, 14, 21, 28, or 35 days after fracture. Fractured tibiae were disarticulated from the knee, and the surrounding muscles were removed. Harvested tissue samples were placed in 10% neutral buffered formalin for 3 days and then decalcified using EDTA at a 14% concentration and pH 7.3. The tissues were automatically infiltrated in the processing machine and manually embedded in paraffin. Alcian blue hematoxylin/orange G staining was done to visualize cartilage and bone, respectively. Histomorphometric analyses were performed using the WinROOF software program, ver. 5.6.0 (Mitani Corp., Tokyo, Japan) to determine the area of bone and cartilage in the external fracture callus by tracing. At least three non-consecutive sections were used for histomorphometric analyses, and the mean of three represented one sample. Four specimens were included in each group for analyses. The mean from four samples was used in statistical analyses to determine the composition of the fracture callus. Cortical bone and internal calluses were excluded from the histomorphometric analyses. In addition, tartrate-resistant acid phosphatase (TRAP) staining was performed for the sections using a TRAP-staining kit (Cosmo Bio Co., LTD., Tokyo, Japan), and counterstained with Fast Green FCF. The mean numbers of TRAP-positive cells in external and internal calluses for three sections in each specimen were manually calculated using the WinROOF software program, ver. 5.6.0 (Mitani Corp.). The mean numbers among four different samples at each time point were analyzed.

### Real-time reverse transcription polymerase chain reaction

For RNA analyses, mice were sacrificed on days 7, 10, 14, 21, 28, or 35 following surgery. Fracture calluses including cortical bone were carefully dissected free of soft tissue. The bone marrow was flushed out using phosphate-buffered saline, and the samples were immediately snap-frozen in a liquid nitrogen bath. Frozen tissue samples were smashed using the TissueLyser system (Qiagen, Hilden, Germany), and total RNA was purified via phase separation using the RNeasy Lipid Tissue Mini Kit (Qiagen) according to the manufacturer’s protocol. Total RNA (1 μg) per callus was reverse transcribed to make single-stranded cDNA using the SuperScript III First-Strand Synthesis System (Invitrogen, Carlsbad, CA, USA). Polymerase chain reaction (PCR) was performed using predesigned FAM dye-labeled TaqMan MGB probe and primer sets (Applied Biosystems, Foster City, CA, USA) in a StepOnePlus Real-Time PCR system (Applied Biosystems). The following mouse specific primers were used for the assessment: *Col2a1*; Mm01309565_m1, *Col10a1*; Mm00487041_m1, *Bglap*; Mm03413826_mH, *Acp5*; Mm00475698_m1, *Gapdh*; Mm99999915_g1. All genes were normalized with *Gapdh*. The mean numbers among four different samples at each time point were analyzed.

### Mechanical testing

The mechanical properties of the fractured tibiae were evaluated using a three-point bending machine (MZ-500S; Maruto, Co., Ltd., Tokyo, Japan). Samples were positioned so that the loading point was at the fracture site. A load speed of 5 mm/min with a 500-N load cell was applied midway between two supports placed 6–8 mm apart. The maximum load and stiffness were calculated using the CTRwin software program, ver. 1.05 (System Supply Co., Ltd., Kanagawa, Japan). We used five specimens for each group.

### Statistical analyses

Data were presented as mean ± standard error (SEM). Fisher’s exact test was used for the radiographic assessment of union and delayed union rates. Mann-Whitney U test was used for the data from micro-CT and biomechanical, histomorphometoric, and gene expression analyses. These tests were performed using the XLSTAT software program (Mindware Inc., Okayama, Japan), with *P* values < 0.05 considered statistically significant.

## Electronic supplementary material

Supplemental tables
