# Supplementary material for: Continuous infusion of PTH1–34 delayed fracture healing in mice
Source: Sci Rep. 2018 Sep 4;8:13175. doi: 10.1038/s41598-018-31345-1 (PMC6123430; doi:10.1038/s41598-018-31345-1)
Supplement: Supplementary file 1 — Supplemental tables [file 41598_2018_31345_MOESM1_ESM.pdf]

Continuous infusion of PTH_{1-34} delayed fracture healing in mice

Kiminori Yukata, Tsukasa Kanchiku, Hiroshi Egawa, Michihiro Nakamura, Norihiro Nishida, Takahiro Hashimoto, Hiroyoshi Ogasa, Toshihiko Taguchi, Natsuo Yasui

Supplemental Table 1. Histomorpometric analyses for the contralateral tibiae after two weeks' treatment.

	Cont. control	Cont. PTH	Int. control	Int. PTH
T.Ar (mm^2)	1.78±0.08	1.63±0.04	1.89±0.06	1.65±0.10
BV/TV (%)	13.40±0.57	12.76±1.50	**9.91±0.37**	**13.36±1.11***
Tb.Th (μm)	37.43±1.33	37.35±2.25	**30.76±0.41**	**41.98±1.00***
Tb.N (N/mm)	3.58±0.07	3.38±0.24	3.22±0.09	3.18±0.24
Tb.Sp (μm)	242.36±5.55	264.67±23.22	280.99±8.77	279.93±24.66
OV/BV (%)	2.73±0.51	5.51±1.58	**1.15±0.33**	**3.36±0.20***
OS/BS (%)	17.97±2.65	26.22±4.78	**7.47±1.72**	**24.99±1.06***
O.Th (μm)	2.83±0.13	3.80±0.55	**2.30±0.14**	**2.88±0.09***
Ob.S/BS (%)	16.13±2.23	26.75±4.65	**8.02±1.83**	**29.52±1.77***
ES/BS (%)	**8.99±0.64**	**14.02±1.32***	**5.93±0.59**	**11.65±0.54***
N.Oc/B.Pm (N/100mm)	**334.76±26.73**	**529.60±40.28***	**276.69±20.56**	**458.08±39.39***
Oc.S/BS (%)	**4.47±0.55**	**6.99±0.61***	**3.06±0.34**	**5.68±0.41***
MAR (μm/day)	**1.70±0.04**	**2.99±0.19***	**1.29±0.07**	**2.95±0.08***
MS/BS (%)	35.05±1.55	35.96±1.63	**24.41±2.73**	**37.30±0.83***
BFR/BS (μm^3/μm^2/y)	**217.31±9.29**	**395.18±37.89***	**116.23±16.15**	**401.28±8.41***
sL.S/BS (%)	**25.86±0.96**	**32.49±2.05***	**20.90±2.59**	**30.36±1.59***
dL.S/BS (%)	22.11±1.59	19.72±1.39	**13.96±1.74**	**22.12±1.12***

Supplemental Table 2. Micro-CT analysis for the contralateral tibiae after two weeks' treatment.

A. Metaphysis at the Proximal Tibia

	Continuous Control	Continuous PTH	Intermittent control	Intermittent PTH
**Cortical BMD** (mg/cm^3)	929.18 ± 5.7	**778.38 ± 16.5***	905.18 ± 19.2	938.98 ± 6.5
**Trabecular BMD** (mg/cm^3)	438.98 ± 30.3	450.38 ± 12.2	377.08 ± 19.2	**531.78 ± 11.8***
**Total BMD** (mg/cm^3)	502.08 ± 14.1	467.5 ± 10.0	500.08 ± 16.4	**568.2 ± 6.1***
**Cortical bone thickness** (cm)	0.01028 ± 0.0001	**0.00914 ± 0.0001***	0.01018 ± 0.0002	0.01069 ± 0.0002
**Cortical bone ratio** (%)	27.6 ± 0.4	**24.2 ± 0.7***	27.3 ± 1.0	28.6 ± 0.9
**Trabecular bone ratio** (%)	52.1 ± 2.6	59.4 ± 0.4	62.8 ± 3.0	**54.6 ± 1.8***
**Minimum 2nd moment of area** (mg.cm)	0.0358 ± 0.0029	0.0340 ± 0.0015	0.0376 ± 0.0019	**0.0462 ± 0.0028***
**Pola area moment of inertia** (mg.cm)	0.0855 ± 0.0082	0.0793 ± 0.0034	0.0838 ± 0.0043	**0.1033 ± 0.0066***
**All bone area** (cm^2)	0.0257 ± 0.0011	0.0267 ± 0.0007	0.0259 ± 0.0008	0.0272 ± 0.001
**All bone volume** (cm^3)	0.00259 ± 0.00011	0.00269 ± 0.00007	0.00261 ± 0.00008	0.00274 ± 0.00011
**All bone mineral content** (mg)	1.306 ± 0.089	1.259 ± 0.039	1.301 ± 0.044	**1.558 ± 0.055***

B. Diaphysis of the Tibia

	Continuous Control	Continuous PTH	Intermittent control	Intermittent PTH
**Cortical BMD** (mg/cm^3)	1275.8 ± 7.4	**1242.1 ± 7.0***	1270.8 ± 6.7	1263.1 ± 3.1
**Trabecular BMD** (mg/cm^3)	368.9 ± 6.6	**320.8 ± 1.9***	358.0 ± 2.1	363.0 ± 5.0
**Total BMD** (mg/cm^3)	921.8 ± 15.9	**800.9 ± 9.6***	908.6 ± 3.3	918.0 ± 14.2
**Cortical bone thickness** (cm)	0.0197 ± 0.001	0.0167 ± 0.0004	0.0195 ± 0.0003	0.0203 ± 0.0005
**Cortical bone ratio** (%)	61.0 ± 1.6	**52.1 ± 0.8***	60.3 ± 0.3	61.7 ± 1.3
**Trabecular bone ratio** (%)	52.5 ± 0.8	**47.3 ± 0.8***	49.9 ± 0.8	**52.2 ± 0.4***
**Minimum 2nd moment of area** (mg.cm)	0.00794 ± 0.0005	0.00861 ± 0.0006	0.00802 ± 0.0003	0.00845 ± 0.0005
**Pola area moment of inertia** (mg.cm)	0.0189 ± 0.0011	0.0203 ± 0.0013	0.0192 ± 0.0009	0.0202 ± 0.0012
**All bone area** (cm^2)	0.0101 ± 0.0003	0.0109 ± 0.0004	0.0102 ± 0.0004	0.0105 ± 0.0003
**All bone volume** (cm^3)	0.00102 ± 0.00003	0.00110 ± 0.00004	0.00103 ± 0.00003	0.00106 ± 0.00003
**All bone mineral content** (mg)	0.943 ± 0.039	0.882 ± 0.029	0.938 ± 0.021	0.971 ± 0.023
